# Non-Thermal Plasma vs. Low-Level Laser Therapy for Recurrent Oral Ulcers: A Randomized Controlled Pilot Study

**DOI:** 10.3390/biomedicines14010141

**Published:** 2026-01-10

**Authors:** Norma Guadalupe Ibáñez-Mancera, Régulo López-Callejas, Víctor Hugo Toral-Rizo, Benjamín Gonzalo Rodríguez-Méndez, Edith Lara-Carrillo, Rosendo Peña-Eguiluz, Antonio Mercado-Cabrera, Raúl Valencia-Alvarado, Diego Medina-Castro

**Affiliations:** 1 Interdisciplinary Center of Health Sciences Unidad Santo Tomás, Instituto Politécnico Nacional, Ciudad de México 11340, Mexico; nibanezm@ipn.mx; 2 Plasma Physics Laboratory, National Institute of Nuclear Research, Carretera México Toluca S/N, Ocoyoacac 52750, Estado de México, Mexico; regulo.lopez@inin.gob.mx (R.L.-C.); rosendo.eguiluz@inin.gob.mx (R.P.-E.); antonio.mercado@inin.gob.mx (A.M.-C.); raul.valencia@inin.gob.mx (R.V.-A.); diego.medina@inin.gob.mx (D.M.-C.); 3 Orocentro Clinic, Faculty of Dentistry, Autonomous University of the State of Mexico, Av. Paseo Tollocan esq. Jesús Carranza, Toluca de Lerdo 50130, Estado de México, Mexico; vhtoralr@uaemex.mx; 4 Department of Orthodontics, Center of Research and Advanced Studies in Dentistry “Dr Keisaburo Miyata”, Faculty of Dentistry, Autonomous University of the State of Mexico, Av. Paseo Tollocan esq. Jesús Carranza, Toluca de Lerdo 50130, Estado de México, Mexico; elarac@uaemex.mx

**Keywords:** recurrent oral ulcers, non-thermal plasma, low-level laser therapy, placebo

## Abstract

**Background/Objectives**: Recurrent oral ulcers (ROUs) are a common condition that significantly impacts patients’ quality of life. This pilot study was conducted to evaluate the feasibility and preliminary results of using non-thermal plasma (NTP) compared to low-level laser therapy (LLLT) and placebo to treat these ulcers. **Methods**: A prospective, controlled, randomised, parallel-group pilot study was conducted using a convenience sample of 50 patients with ROUs. Patients were randomly assigned (2:2:1) to one of three groups: NTP (*n* = 20), LLLT (*n* = 20), and placebo (*n* = 10). Feasibility and preliminary data acquisition were the primary goals. Exploratory outcomes included ulcer size reduction and safety profile. This was a single-blinded trial, where participants and outcome assessors were masked to group assignment. Ulcer size, pain perception, and time to complete healing were measured. For statistical analysis, ANOVA was used, with a *p*-value ≤ 0.05. **Results**: The groups were comparable at baseline. Exploratory results suggest that NTP demonstrated a promising trend in accelerating healing, with a mean healing time difference of 5.5 days compared to LLLT (2.5 ± 1.9 days vs. 8.0 ± 4.3 days) and 7.1 days compared to placebo (2.5 ± 1.9 days vs. 9.6 ± 5.3 days) (*p* < 0.001). Regarding pain, NTP provided significant and sustained relief. Patients in the NTP group were asymptomatic on day 2, unlike the LLLT and placebo groups, where pain persisted significantly (NTP VAS score at 1 h: 1.1 ± 2.1 vs. LLLT/Placebo VAS score at 1 h: 3.4 ± 2.4 and 7.3 ± 1.9, respectively) (*p* < 0.001). NTP was well tolerated, and no adverse events were reported. **Conclusions**: This pilot study suggests that NTP is a potentially safe and effective therapy for recurrent oral ulcers. Preliminary results indicate that it may accelerate healing and offer superior pain relief, warranting a large-scale clinical trial to confirm these findings.

## 1. Introduction

Oral ulcers (OUs) are a prevalent and complex pathology of the oral mucosa, defined by a discontinuity in the epithelium that exposes the underlying connective tissue. This condition is more than just a local discomfort; it clinically manifests as intense, persistent pain that significantly interferes with essential functions like eating, swallowing, and speaking, profoundly compromising a patient’s quality of life [1,2,3].

The causes are multifaceted, ranging from acute mechanical trauma and infectious agents (viruses, bacteria, fungi) to immunological dysregulation, autoimmune diseases, hypersensitivity reactions, and neoplastic processes [4,5,6]. Among these diverse presentations, recurrent oral ulcers (ROUs) are one of the most common. It’s characterized by several episodes per year of excruciating ulcers that can take up to 30 days to heal, depending on their size [7].

Despite the diversity of available therapeutic approaches, from palliative measures and topical agents such as anaesthetics, barrier protectants, and antiseptics to systemic pharmacological treatments such as corticosteroids and immunosuppressants, many have inherent limitations. These limitations include variable efficacy, undesirable side effects, whether local or systemic, or prolonged recovery times, which often frustrate both patients and clinicians in their search for an effective and lasting resolution [8,9,10].

This clinical reality underscores the urgent need to explore and validate new therapeutic strategies that offer superior, or at least comparable, results, but with an optimised safety profile and faster lesion resolution.

In the context of the search for therapeutic innovation, non-thermal plasma (NTP) has emerged as a cutting-edge biomedical technology, generating growing interest and demonstrating significant potential in various medical specialities, including dermatology for wound healing and the control of skin infections, and particularly prominently in recent years in dentistry [11,12,13]. NTP is defined as a partially ionised gas that operates at near-ambient temperatures. This crucial characteristic confers biocompatibility and allows direct and safe application to sensitive biological tissues, such as the oral mucosa, without inducing thermal damage. Its composition is a complex and dynamic matrix of reactive species, including high-energy electrons, charged ions, a vast array of free radicals, reactive oxygen and nitrogen species (RONS), including ^•^OH, O_3_, NO, NO_2_, O_2_^−^, H_2_O_2_, and low-intensity ultraviolet radiation [14,15,16]. These bioactive species confer on NTP a spectrum of desirable therapeutic properties that make it an excellent candidate for ulcer management: potent broad-spectrum antimicrobial activity (with bactericidal, virucidal, and fungicidal effects), the ability to modulate the local inflammatory response (reducing edema and erythema), and the active promotion of pro-angiogenic and tissue remodelling processes essential for wound repair [17,18].

The hypothesis underlying the application of NTP in oral ulcers lies in its multifaceted and finely regulated mechanism of action at the cellular and molecular levels. Recent scientific evidence has shown that NTP can induce selective apoptosis in pathogenic cells, such as bacteria, or dysfunctional and damaged cells, while simultaneously stimulating the proliferation and migration of cells essential for tissue regeneration, such as fibroblasts and keratinocytes [19,20,21]. This duality of action, i.e., the selective elimination of harmful elements and the stimulation of repair, could clinically translate into an acceleration of healing processes, an effective reduction of the microbial load in the ulcer bed and, consequently, a decrease in pain and a faster recovery, critical aspects for a favourable clinical resolution in the oral cavity.

On the other hand, low-level laser therapy (LLLT), also known as photobiomodulation, has been established as a safe therapeutic modality for managing various oral lesions, including recurrent and traumatic oral ulcers [22,23,24]. Lasers can modulate cellular and tissue responses by absorbing photons by cellular chromophores, mainly cytochrome c oxidase. Its mechanisms of action include the promotion of adenosine triphosphate (ATP) synthesis, which supplies energy for repair processes; the stimulation of cell proliferation, promoting re-epithelialisation; the induction of angiogenesis, improving vascularisation and nutrient supply; and an effective modulation of inflammation, reducing pro-inflammatory mediators [25]. Furthermore, certain types of lasers possess direct or indirect antimicrobial effects, for example, through the generation of RONS or localised thermal effects, which contribute to the decontamination of the lesion site, a crucial factor in the healing of oral ulcers [26,27].

Since both NTP and LLLT offer promising mechanisms for accelerated healing and infection control in oral ulcers, it is crucial to rigorously compare them with an appropriate placebo group to discern their true efficacy. The inclusion of a placebo group is essential in the design of randomised controlled clinical trials. This group allows us to differentiate the actual effect of the active intervention from the placebo effect, which is a clinical response attributable to psychological or non-treatment-specific factors, or from the spontaneous resolution of the pathology, which is common in many oral ulcers [28,29,30]. A carefully designed placebo isolates the specific impact of the proposed treatment, providing a rigorous and ethically justifiable baseline for assessing the true efficacy and safety profile of new therapies.

Table 1 provides a structured summary of the main investigations and concepts supporting the potential of NTP and LLLT in treating oral ulcers to contextualise the need for this study clearly. Therefore, the primary objective of this pilot study was to evaluate the feasibility of the methodology and obtain preliminary data on the clinical efficacy and safety profile of NTP. Specifically, its impact on pain reduction and healing time in ROUs was analysed, contrasting it with LLLT and a placebo group. This is the first randomised, controlled pilot trial to directly compare NTP with LLLT and a placebo to treat recurrent oral ulcers. The ultimate goal is that the findings of this study will justify a larger-scale clinical trial to confirm the results and lay the groundwork for future clinical guidelines and dental management protocols.

## 2. Materials and Methods

### 2.1. Study Design and Trial Registration

A prospective, randomised, controlled pilot study was conducted to evaluate the feasibility of the methodology and obtain preliminary data on the safety and efficacy profile of non-thermal plasma, low-level laser therapy, and placebo in the treatment of recurrent oral ulcers. The study protocol was reviewed and approved by the Research and Ethics Committee of the Faculty of Dentistry of the Autonomous University of the State of Mexico (Registration Number: CEICIEAO-2018-001). Participants were recruited at the Orocentro Dental Clinic of the Autonomous University of the State of Mexico.

The trial was conducted in accordance with the Declaration of Helsinki (2013 version) and recognising the importance of pre-registration for all randomised controlled trials, as mandated by the CONSORT guidelines. However, given the exploratory nature and limited sample size of this specific pilot study, whose primary objective was to assess feasibility and optimal parameters rather than definitive efficacy, the team did not register the protocol a priori in an international public clinical database. The ethical oversight provided by the Research and Ethics Committee of the Faculty of Dentistry of the Autonomous University of the State of Mexico (Registration Number: CEICIEAO-2018-001) served as the primary and sufficient regulatory framework for the ethical conduct of this specific preliminary investigation. Still, the research team is committed to prospectively registering all future efficacy trials.

### 2.2. Protocol Access

The complete protocol is available upon reasonable, justifiable request addressed to the corresponding author, who will initiate the necessary approval process with the Institutional Ethics Committee. Access is subject to the final approval of this body due to internal document management policies.

### 2.3. Study Sample and Feasibility

A total of 50 patients were included in this study, with an allocation ratio of 2:2:1 (NTP: *n* = 20; LLLT: *n* = 20; Placebo: *n* = 10). The sample size was not determined a priori based on power calculations for efficacy, as the primary objective of this exploratory pilot study was to assess recruitment feasibility for a future large-scale trial and to obtain preliminary data on clinical outcomes. The number of participants was determined by convenience sampling based on the recruitment rate over a six-month period, which is consistent with published recommendations for pilot feasibility trials. We confirm that this size was sufficient to assess the safety profile and the viability of the intervention procedures. A post hoc analysis was performed (results are discussed in the Section 4) to confirm that the sample size was sufficient to detect the clinically significant differences observed in the NTP group compared to the other interventions.

### 2.4. Selection Criteria

Inclusion criteria: This study included patients over 18 years of age with a clinical diagnosis of ROUs lasting no more than 48 h. Patients must have no evidence of malignancy in the lesion and must have provided written informed consent before participation.

Exclusion criteria: Patients with immunodeficiencies and uncontrolled systemic diseases, such as decompensated diabetes mellitus, as well as pregnant or breastfeeding women, were excluded. Patients receiving concomitant systemic corticosteroids or chemotherapy, and those with oral ulcers of obvious traumatic origin caused by foreign objects or sharp dental edges that could not be removed before treatment, were also excluded.

### 2.5. Randomization and Blinding

Participants were randomly assigned to one of three treatment groups (NTP, LLLT, or placebo). Allocation concealment was ensured by placing the treatment codes in opaque, sealed, and sequentially numbered envelopes that were opened only after each participant met the inclusion criteria and signed the informed consent.

Due to the operational nature and specific physical characteristics of the NTP and LLLT devices, it was not feasible to blind the participants or the treating clinician to the active intervention. Therefore, this study was conducted as an open-label trial with respect to treatment administration.

Crucially, this study maintained outcome assessor blinding. All outcome measurements, including pain assessment (VAS), ulcer size measurement, and healing time determination, were performed by a single, trained, and independent assessor, who was separate from the treating clinician and fully masked to each participant’s group assignment. This approach was used to minimize detection bias and ensure the objectivity and validity of the primary efficacy data.

**Figure 1:**
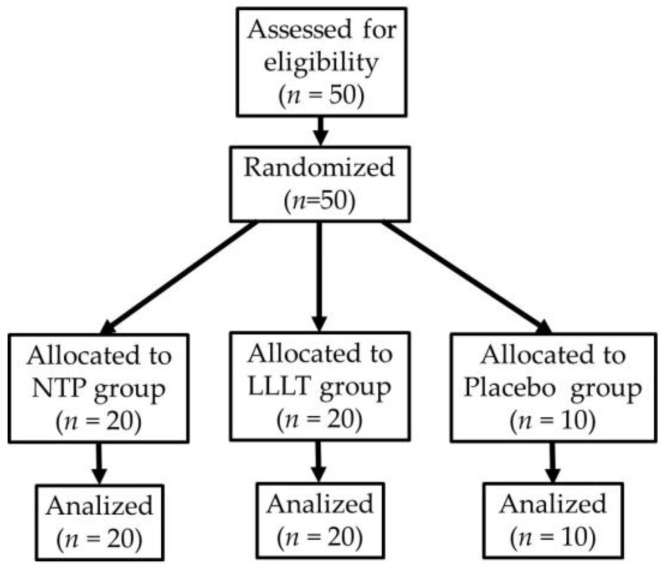
Flow diagram of participant recruitment and allocation (CONSORT).

Figure 1 illustrates participant recruitment, random assignment, and follow-up to ensure transparency and reproducibility of the pilot study.

### 2.6. Non-Thermal Plasma (NTP) Application

NTP treatment was administered using a needle-type dielectric barrier discharge (DBD) device. Optimised for biomedical applications, this equipment was designed and built at the National Institute of Nuclear Research (ININ) in Mexico. The device was operated at a power of 20 W and a frequency of 13.56 MHz, using helium as the working gas and maintaining a constant and controlled flow of 0.5 L/min.

This configuration resulted in an irradiance of 0.5 W/cm^2^ directed at the ulcer tissue. Importantly, this irradiance remained within the safety limits established by the International Commission on Non-Ionising Radiation Protection (ICNIRP), whose safe threshold is 4 W/cm^2^, thus ensuring the integrity and safety of the patient’s tissue [31].

The application was performed by keeping the applicator tip at a constant distance of 5 mm from the ulcer surface to ensure the uniform dispersion and optimal distribution of reactive plasma species. During the procedure, an infrared detection sensor monitored the temperature rise in the tissue in real time, ensuring that the surface temperature remained within a safe range of 28 °C to 30 °C. This thermal control is crucial to prevent cell damage and optimise therapeutic outcomes.

Two applications of NTP, lasting three minutes each, were administered to each ulcer. A one-hour interval was established between the first and second applications to assess the initial tissue response. During the procedure, a saliva ejector and sterile gauze were used to keep the treatment area as dry as possible, a critical factor in maximising the plasma’s efficacy. The beam was ensured to cover the entire lesion and leave a 1–2 mm safety margin of surrounding healthy tissue to treat areas of inflammation or peripheral contamination.

### 2.7. Low-Level Laser Therapy (LLLT) Application

The intervention for the comparison group consisted of low-level laser therapy using the Quantum^®^ Therapeutic Laser IR-810 device (Laser Systems, Querétaro, Mexico), designed for dental use. As a non-thermal therapy, its safety is based on the absence of a significant increase in tissue temperature [32,33]. The application protocol followed the manufacturer’s recommendations for managing recurrent oral ulcers. Three treatment sessions were administered to each ulcer to standardise the methodology with the NTP group. The protocol consisted of an initial application, followed by a second session administered one hour later, and a final third session another hour later.

A non-contact scanning technique was used, keeping the applicator tip at a constant distance of 5 mm from the lesion, ensuring complete irradiation of the ulcer. The exposure time was automatically determined according to the device’s preprogrammed aphthous ulcer settings. The technical parameters of the laser used were: a wavelength of 810 nm (infrared emission), an output power of 200 mW in continuous wave (CW) mode, and an energy density of 6 J/cm^2^ per treatment area [34].

### 2.8. Placebo Group

Patients assigned to the placebo group underwent a sham procedure that served as a comparison for the active NTP and LLLT. This group aimed to distinguish the actual effects of the interventions from any spontaneous improvements or psychological influences. The same NTP or laser devices were utilised for the placebo group without activating the plasma or light emission. The devices were turned on solely to display their indicator lights, and the operator manipulated the applicator in the same manner and for the same duration as in the active treatment groups. The applicator was maintained at a consistent distance from the lesion, specifically 5 mm.

Strict blinding protocols were implemented to ensure that both patients and operators assessing clinical outcomes were unaware of group assignments. This was accomplished using modified devices designed to simulate the operation of the active devices, thereby minimising the potential for bias in treatment perception and outcome assessment.

### 2.9. Primary and Secondary Outcomes

Primary Outcomes: Due to the exploratory pilot nature of this study, the primary outcomes focused on feasibility, measured by patient recruitment rates and adherence to the protocol. The preliminary clinical primary outcomes, collected to inform a future definitive trial, were time to complete healing (days) and reduction in pain perception (measured by the Visual Analogue Scale [VAS] score at day 2).

Secondary/Exploratory Outcomes: The secondary outcomes included a reduction in ulcer size (measured in mm), the safety profile (defined by the presence of adverse events and local irritation/inflammation), and the cumulative need for rescue medication.

### 2.10. Assessment and Follow-Up

A standardised clinical assessment of the oral ulcers was performed at the following time points: before the first treatment application (baseline), immediately after the completion of treatment, and during follow-up on days 2 and 7. Parameters assessed included ulcer size using a digital calliper to ensure objectivity, and pain intensity, assessed using a VAS ranging from 0 to 10 (with zero corresponding to no pain). Additionally, irritation, inflammation, or infection was recorded at each visit. Standardised photographs of the lesions were taken at each follow-up visit to document healing progression. Finally, any adverse events or complications reported by patients or detected during clinical assessments were monitored and recorded.

### 2.11. Statistical Analysis

The analysis was performed according to the intention-to-treat (ITT) principle, including all randomised participants. Missing data were assumed to be Missing At Random (MAR) and handled using a complete-case analysis, as the loss-to-follow-up rate was negligible and did not exceed 5% in any group.

A one-way analysis of variance (ANOVA) was used to evaluate and compare quantitative variables between the three groups (NTP, LLLT, and placebo). Normality was assessed using the Shapiro–Wilk test, and homogeneity of variance was assessed using the Levene test; both assumptions were met. In cases where the ANOVA indicated a significant difference, a post hoc Tukey HSD (Honestly Significant Difference) analysis was performed to identify specific differences between groups. The relationship between quantitative variables was assessed using Pearson’s correlation coefficient. For key outcomes (healing time and pain score), 95% confidence intervals (CIs) are used to measure the magnitude of the effect, and *p*-values are also reported. A 95% confidence interval was established in all analyses, and a *p*-value ≤ 0.05 was considered statistically significant.

## 3. Results

The results of the efficacy evaluation are presented with 95% CIs to provide a robust interpretation of the observed effects. A post hoc power analysis confirmed that the pilot study had more than 99% power to detect the observed significant clinical effects. The pilot study included 50 patients diagnosed with ROUs. The total population consisted of 13 men (26%) and 37 women (74%), with an age range of 18 to 68 years and a mean age of 44.4 ± 18.3 years. All ulcers had an initial size between 2 and 15 mm and were located on the mobile mucosa (tongue, cheeks, vestibule, soft palate, and floor of the mouth). At diagnosis, all patients reported a pain level between 7.0 ± 2.1 and 7.2 ± 2.7 on a 0-to-10 VAS.

Participants were randomly assigned to three groups. The NTP group consisted of 20 patients (6 men, 30%; 14 women, 70%), with an age range of 19 to 67 years and a mean of 44.3 ± 17.5 years. The LLLT group included 20 patients (3 men, 15%; 17 women, 85%), with an age range of 18 to 68 years and a mean of 44.5 ± 18.9 years. Finally, the placebo group consisted of 10 patients (4 men, 40%; 6 women, 60%), aged 18 to 67 years, with a mean of 44.4 ± 20.3 years. No statistically significant differences were found between groups regarding age, sex, ulcer size, and pain score at baseline (*p* > 0.05).

Table 2 presents a comparative summary of the clinical variables assessed in the three groups to examine the treatment effect. Ulcer size and pain scores were measured at baseline, i.e., before treatment, one hour after treatment completion, and on days 2 and 7 of follow-up. Time to repair, or the total mucosal regeneration time in days, was recorded until complete healing. Data are presented as mean ± standard deviation, with 95% CI to measure the magnitude of the effect, in addition to *p*-values.

### 3.1. Efficacy in Reducing Ulcer Size and Healing Time

The three groups showed a similar mean initial ulcer size, with no statistically significant differences (*p* = 0.72). However, the preliminary finding of the clinical outcome of tissue regeneration was significantly different between the active treatments and the placebo groups.

**Figure 2:**
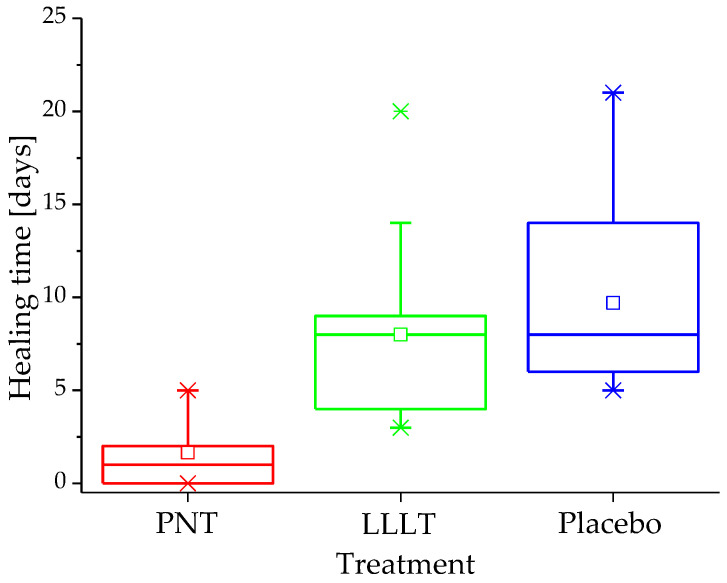
Distribution of ulcer healing time (days) in the study groups.

Ulcer regeneration time underscored this difference. The differences in healing time observed in this pilot study were significant (*p* < 0.001). Figure 2 illustrates the distribution of total healing time across a range of quartiles (box plot). A post hoc analysis revealed that ulcers in the NTP group healed in 2.5 ± 1.9 days (95% CI, 1.6–3.4), which was faster than in the LLLT group (8.0 ± 4.3 days; 95% CI, 6.0–10.0) and placebo groups (9.6 ± 5.3 days; 95% CI, 6.3–12.9) (both *p* < 0.001).

In line with the accelerated healing times, the results also showed that by day 7 of follow-up, 100% of the ulcers in the NTP group had healed entirely. In contrast, only 30% of the ulcers in the LLLT group and 50% of those in the placebo group showed complete healing by day 7.

### 3.2. Impact on Pain Perception

**Figure 3:**
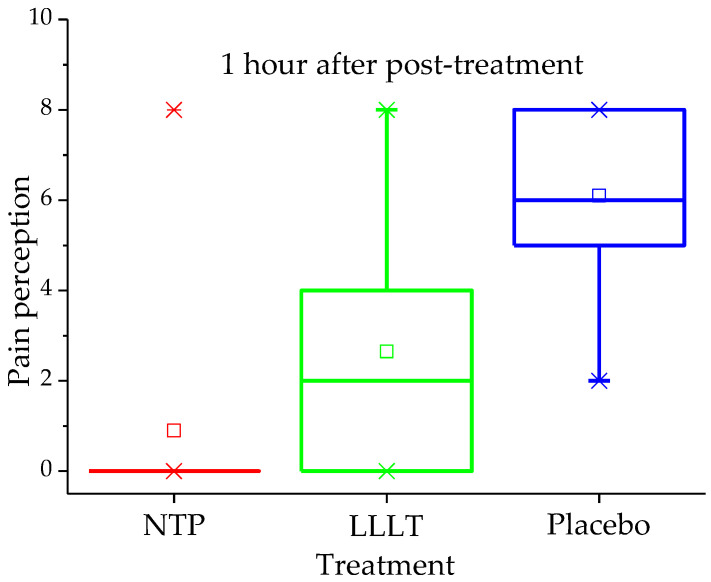
Pain score (VAS 0–10) by treatment group one hour after intervention.

Preliminary analysis of the impact on pain perception also yielded significant results in favour of NTP treatment, as shown in Table 2. The differences in pain scores between the three groups were statistically significant (*p* < 0.001) at all post-treatment assessment points. A key finding of this pilot study was that the pain reduction in the NTP group, with a mean score decreasing from 7.0 ± 2.1 to 1.1 ± 2.1 in just one hour, was greater than that observed in the LLLT and placebo groups (*p* < 0.001 in both cases). The distribution of pain one hour after treatment is clearly illustrated in the quartile plot (Figure 3). Furthermore, all patients in the NTP group were completely asymptomatic on day 2.

In contrast, patients in the LLLT group, who started with a score of 7.2 ± 2.7, experienced an initial decrease to 3.4 ± 2.4 at 1 h, but their pain increased again on day 2, reaching a mean of 4.8 ± 2.6. Moreover, 70% of them still reported pain on day 7. In the placebo group, the pain reduction was minimal and transient, with the mean decreasing only from 7.2 ± 2.7 to 6.3 ± 2.1 at 1 h after sham treatment, and with a slight reduction to 5.8 ± 2.6 on day 2. Fifty per cent of these patients continued to report pain on day 7.

## 4. Discussion

The preliminary findings of this clinical study support the hypothesis that non-thermal plasma (NTP) represents a potentially superior therapeutic modality for managing recurrent oral ulcers. It outperformed both low-level laser therapy (LLLT) and the placebo group. Our results demonstrate a significant acceleration in healing and more effective and sustained pain relief with NTP treatment.

The superiority of NTP in accelerating healing is a finding of significant clinical relevance. Healing time was significantly shorter in the NTP group compared to the LLLT and placebo groups (*p* < 0.001, Table 2). Specifically, the mean healing time of 2.5 days observed with NTP is substantially shorter than the 7 to 14 days typically reported in the literature for spontaneous resolution of recurrent oral ulcers, and faster than the 5 to 10 days commonly observed with other active therapies such as conventional LLLT protocols or topical corticosteroids.

These initial results surpass evidence from other studies indicating that LLLT shortens healing time compared to placebo, but not at the speed levels observed with NTP [35,36,37]. This effect could be attributed to NTP’s multifactorial ability to induce fibroblast and keratinocyte proliferation, while modulating the inflammatory cascade and exerting a potent antimicrobial effect on the ulcer bed [38,39,40]. Unlike LLLT, whose mechanism of action focuses on photobiomodulation at the cellular level through specific chromophores, NTP acts through a broad range of RONS that can penetrate the cell membrane and exert their biological effects more directly and forcefully on the microbial burden and the activation of tissue repair mechanisms.

The impact of NTP on pain reduction was also notable. While patients treated with LLLT and placebo showed significant pain persistence, all patients in the NTP group were reported to be asymptomatic on day 2. Pain relief observed in the NTP group showed a dramatic reduction on the VAS scale in just 1 h, a change that was significantly superior to that of the other two groups (*p* < 0.001, Table 2). A notable finding in this pilot study in the LLLT group was the initial decrease in pain at 1 h, followed by an increase on day 2, suggesting that its analgesic and anti-inflammatory effects may be more transient and fail to modulate the long-term inflammatory response. In contrast, the rapid and complete elimination of pain with NTP could be related to its ability to neutralise pro-inflammatory mediators and its antimicrobial effect [41,42], which reduces inflammation and irritation in the exposed tissue.

One notable advantage of NTP, particularly relevant to clinical practice, is the efficiency of its application protocol. NTP treatment requires only two sessions, compared to the three sessions typically needed for LLLT. This reduced number of applications makes the procedure more convenient for the patient and the clinician. Overall, it represents a significant improvement in efficiency and decreased clinical chair time.

No significant adverse effects were observed regarding treatment safety. Patients in the NTP group reported excellent tolerance to the procedure, presenting no signs of irritation, inflammation, infection, or other complications related to plasma application. This favourable safety profile is crucial for considering NTP as a viable option in clinical practice.

Despite these promising results, we must acknowledge some limitations of our study. One inherent limitation of our study design is the inability to achieve participant and treating clinician blinding. This was an unavoidable consequence of the distinct operational and physical characteristics of the NTP and LLLT devices, resulting in an open-label design for the intervention phase. This methodological constraint introduces a potential risk of performance bias. However, this risk was substantially mitigated by maintaining outcome assessor blinding. Since all primary efficacy measures (pain, ulcer size, and healing time) were objectively collected by an independent, masked clinician, we ensured the objectivity of the measurements and minimized the risk of detection bias in the final reported outcomes. The unequal sample size between groups, particularly a smaller placebo group, was a limitation. This specific sample size was selected because the study was a pilot designed to assess recruitment feasibility and methodology. This decision was made because the primary objective of the placebo group was to serve as a control to isolate the actual effect of the active interventions, without exposing a larger number of patients than necessary to a treatment not expected to have a therapeutic effect. Moreover, a post hoc power analysis confirmed that the sample size was sufficient to detect the observed statistically significant differences. Furthermore, the 74% female majority in our sample may limit the generalizability of our findings to the general population, as aetiology and treatment response could differ between sexes.

Another limitation is the relatively short follow-up period. While our pilot study focused on short-term efficacy in the acute phase of ulcers, a more extended observation period would be crucial to assess long-term safety and, more importantly, the recurrence rate [43,44,45]. Furthermore, while LLLT has shown positive outcomes in accelerating healing in many studies, recent literature continues to confirm its efficacy in reducing pain and promoting lesion remission [46]. Future clinical trials should address these issues by including a larger, more balanced sample and a more extended follow-up period.

## 5. Conclusions

This pilot study suggests that NTP could be a superior and highly effective therapeutic modality for treating recurrent oral ulcers. The results demonstrated that NTP treatment significantly accelerated healing time, achieving complete healing in an average of 2.5 ± 1.9 days, notably faster than in the LLLT and placebo groups. Similarly, NTP provided rapid and sustained pain relief, with patients reporting a complete absence of pain on the second day of follow-up, in contrast to the persistent pain observed in the other two groups. Additionally, the efficiency of NTP treatment, which required only two applications compared to three for LLLT, makes it a more convenient and time-effective option for clinicians and patients. Furthermore, the treatment demonstrated an excellent safety profile, with no significant adverse effects reported. The compelling results of this pilot study warrant a larger-scale randomised controlled clinical trial to confirm the potential of NTP as a first-line treatment for this condition.

## Figures and Tables

**Table 1:** Comparative summary of the relevant literature.

Focus/Study Type	Therapeutic Technology	Key Findings	References
Nature of ROUs	N/A	A common condition that affects quality of life, causes pain, and hinders essential functions like eating and speaking.	[1,2,3]
Aetiology and pathogenesis	N/A	Complex and multifactorial aetiology, including trauma, infectious agents, and immunological irregularities.	[4,5,6]
Recurrent aphthous stomatitis	N/A	One of the most common clinical presentations is recurring episodes that can take up to 30 days to heal.	[7]
Limitations of existing therapies	N/A	Variable efficacy, undesirable side effects, and long recovery times highlight the need for new therapies.	[8,9,10]
Potential of NTP	Topical agents and systemic treatments (corticosteroids, immunosuppressant).	Emerging as a cutting-edge biomedical technology in various specialities, including dermatology and dentistry.	[11,12,13]
Mechanism of action of NTP	NTP	Described as a partially ionized gas that acts through RONS, it is biocompatible and safe for sensitive tissues.	[14,15,16]
Therapeutic properties of NTP	NTP	Possesses broad-spectrum antimicrobial activity, modulates the inflammatory response, and promotes tissue regeneration.	[17,18]
Mechanisms of action of NTP at the cellular level	NTP	Induces selective apoptosis in pathogens and damaged cells while stimulating the proliferation of regenerative cells like fibroblasts and keratinocytes.	[19,20,21]
Use and efficacy of LLLT	NTP	A well-established and safe therapy for managing various oral lesions, including ulcers.	[22,23,24]
Mechanisms of action of LLLT	LLLT	Photobiomodulation promotes ATP synthesis, cell proliferation, and angiogenesis and modulates inflammation.	[25]
Antimicrobial effects of LLLT	LLLT	Has a direct and indirect antimicrobial effect, contributing to the decontamination of the lesion site.	[26,27]
Importance of the placebo group	LLLT	Essential for distinguishing the intervention’s actual effect from psychological effects or spontaneous healing.	[28,29,30]

N/A = Data not available.

**Table 2:** Comparison of clinical parameters between groups.

Parameter	Evaluation Time	NTP (*n* = 20)	LLLT (*n* = 20)	Placebo (*n* = 10)	*p*-Value
Ulcer size [mm]	Pre-treatment	6.1 ± 3.6 ^1^ (4.4–7.8)	6.8 ± 3.9 ^1^ 5.0–8.6)	6.0 ± 3.7 ^1^ (3.4–8.6)	0.72
	1 h post-treatment	3.1 ± 3.4 ^1^ (1.5–4.7)	5.3 ± 3.2 ^1^ (3.8–6.8)	5.8 ± 3.2 ^1^ (3.9–7.7)	<0.01
	Day 2	1.4 ± 1.8 ^1^ (0.6–2.2)	3.4 ± 2.7 ^1^ (2.1–4.7)	5.2 ± 2.6 ^1^ (3.6–6.8)	<0.001
	Day 7	0.0 ± 0.0 ^1^ (0.0–0.0)	2.5 ± 2.3 ^1^ (1.4–3.6)	1.7 ± 2.1 ^1^ (0.4–3.0)	<0.001
Healing time [days]		2.5 ± 1.9 ^1^ (1.6–3.4)	8.0 ± 4.3 ^1^ (6.0–10.0)	9.6 ± 5.3 ^1^ (6.3–12.9)	<0.001
Pain score [VAS, 0–10]	Pre-treatment	7.0 ± 2.1 ^1^ (6.0–8.0)	7.2 ± 2.5 ^1^ (6.0–8.4)	7.2 ± 2.7 ^1^ (5.5–8.9)	0.95
	1 h post-treatment	1.1 ± 2.1 ^1^ (0.1–2.1)	3.4 ± 2.4 ^1^ (2.3–4.5)	6.3 ± 2.1 ^1^ (5.0–7.6)	<0.001
	Day 2	0.0 ± 0.0 ^1^ (0.0–0.0)	4.8 ± 2.6 ^1^ (3.6–6.0)	5.8 ± 2.6 ^1^ (4.2–7.4)	<0.001
	Day 7	0.0 ± 0.0 ^1^ (0.0–0.0)	2.2 ± 2.2 ^1^ (1.2–3.2)	3.1 ± 3.2 ^1^ (1.1–5.1)	<0.01

^1^ mean ± SD, (CI) 95% confidence interval.

## Data Availability

All data generated or analysed during this study are included in this published article. However, in compliance with institutional regulations on patient confidentiality and data management policies, the complete raw, anonymised individual participant data (IPD) underlying the results of this article are available upon reasonable request addressed to the corresponding author. Access will be granted upon a formal data-sharing agreement, approval by the Institutional Ethics Committee, and confirmation of the request’s justification. Data access will be subject to a strict protocol of data anonymisation and will not be granted for commercial purposes.

## Abbreviations

The following abbreviations are used in this manuscript:

ROUs	Recurrent oral ulcers
LLLT	Low-level laser therapy
NTP	Non-thermal plasma
RONS	Reactive oxygen and nitrogen species
ICNIRP	International Commission on Non-Ionising Radiation Protection
ATP	Adenosine triphosphate
DBD	Dielectric barrier discharge
VAS	Visual analog scale
